# The effects of early life adversity on children’s mental health and cognitive functioning

**DOI:** 10.1038/s41398-022-02001-0

**Published:** 2022-06-10

**Authors:** Mark Wade, Liam Wright, Katherine E. Finegold

**Affiliations:** Department of Applied Psychology and Human Development, University of Toronto, Toronto, ON Canada

**Keywords:** Psychology, Psychiatric disorders

## Abstract

Emerging evidence suggests that partially distinct mechanisms may underlie the association between different dimensions of early life adversity (ELA) and psychopathology in children and adolescents. While there is minimal evidence that different types of ELA are associated with specific psychopathology outcomes, there are partially unique cognitive and socioemotional consequences of specific dimensions of ELA that increase transdiagnostic risk of mental health problems across the internalizing and externalizing spectra. The current review provides an overview of recent findings examining the cognitive (e.g., language, executive function), socioemotional (e.g., attention bias, emotion regulation), and mental health correlates of ELA along the dimensions of threat/harshness, deprivation, and unpredictability. We underscore similarities and differences in the mechanisms connecting different dimensions of ELA to particular mental health outcomes, and identify gaps and future directions that may help to clarify inconsistencies in the literature. This review focuses on childhood and adolescence, periods of exquisite neurobiological change and sensitivity to the environment. The utility of dimensional models of ELA in better understanding the mechanistic pathways towards the expression of psychopathology is discussed, with the review supporting the value of such models in better understanding the developmental sequelae associated with ELA. Integration of dimensional models of ELA with existing models focused on psychiatric classification and biobehavioral mechanisms may advance our understanding of the etiology, phenomenology, and treatment of mental health difficulties in children and youth.

## Introduction

Childhood is an important period for the development of socioemotional and cognitive skills given the high degree of brain plasticity that facilitates learning and adaptation to the environment [1, 2]. However, early life adversity (ELA) during periods of heightened plasticity may alter developmental trajectories via complex neurobiological and stress-mediated mechanisms that, in turn, increase the risk of socioemotional and behavioral difficulties in children and youth [3]. ELA encompasses a wide range of experiences such as psychosocial and material neglect, exposure to intimate partner violence, and physical, sexual, and emotional abuse. Some of these experiences involve the *presence of threatening inputs* (e.g., abuse), while others reflect the *absence of expectable inputs* (e.g., neglect). Although the neurodevelopmental consequences of different dimensions of ELA may be at least partially distinct, each of these share a defining feature in that they generally entail a deviation from the expectable environment that requires considerable adaptation by the average child [4]. While this adaptation may foster survival under conditions of stress and adversity, it may be undesirable in more typical developmental circumstances and give rise to a multitude of poor outcomes, including health-risk behaviors that are associated with considerable early morbidity and mortality [5].

ELA is strongly correlated with higher rates of almost all commonly occurring mental health problems, including mood, anxiety, and substance use disorders [6–9]. The association between ELA and mental health difficulties is especially concerning given that more than half of all children and youth will experience at least one form of ELA by adulthood [10, 11], and that ELA accounts for the onset of roughly half of all mental health problems in childhood [6, 10]. Psychiatric difficulties associated with ELA are also more severe, persistent, and treatment-resistant than difficulties and disorders not associated with ELA [7]. Thus, ELA is a critical target of public health inititives designed to reduce population disparities in mental health among children and youth [12].

Recently, there has been somewhat of a paradigm shift towards dimensional models of ELA that aim to better characterize the mechanisms linking particular experiences of adversity to specific developmental outcomes [13–17]. One such model is the Dimensional Model of Adversity and Psychopathology (DMAP) [16, 17], which posits that discrete forms of ELA exist along two broad dimensions: threat and deprivation. In this model, *threat* is operationalized as harm or the threat of harm, while *deprivation* is operationalized as the absence of expected environmental input or complexity. Another dimensional model that is informed by evolutionary life history theory is that of Ellis et al. which conceptualizes ELA along the dimensions of harshness and unpredictability [13]. In this Life History (LH) model, *harshness* is defined as suffering and mortality due to extrinsic forces (i.e., family life/community violence) which are out of the individual’s control, while *unpredictability* is defined as random fluctuations in exposure to harshness. Recent work has integrated the DMAP and LH models to outline three primary dimensions along which specific types of ELA may fall: threat/harshness, neglect/deprivation, and unpredictability [18]. The current review draws on this framework to provide an up-to-date synthesis of the literature focused on evaluating the partially distinct cognitive, socioemotional, and mental health correlates of ELA along each of these three dimensions, while also detailing how alterations in cognitive and socioemotional function serve as a developmental pathway linking ELA to psychopathology in childhood and adolescence. This is important at the current juncture within the literature as it accompanies an ongoing discussion regarding the utility of both DMAP and LH models which may serve as a basis for future ELA research (see McLaughlin et al. for arguments supporting an integrated dimensional approach, and Smith and Pollak and Pollak and Smith for arguments generally opposing such approaches) [18–20].

## Threat/harm

### Threat/harm and psychopathology

Early exposure to threatening/harsh experiences—including physical, sexual and emotional abuse, exposure to domestic violence, or bullying/victimization by peers—is a powerful predictor of later mental health outcomes. Children and adolescents who are exposed to threat have a heightened risk for depression, conduct disorder, substance use, psychosis, self-harm behaviors, and suicide [21–23]. Moreover, recent epidemiological studies demonstrate that children who experience a traumatic event, defined as experiences that are harmful and involve threatened death or violence, are two times more likely to develop a psychiatric disorder than non-trauma exposed children [24]. Additionally, one in six children who experience a traumatic event meet criteria for post-traumatic stress disorder (PTSD) [25], a disorder that only occurs in individuals who have experienced trauma. It is also important to note that threat, as defined within the DMAP, is generally focused on repeated interpersonal harm (e.g., abuse or community violence), and that this form of ELA is more strongly associated with poor mental health outcomes in children and youth than non-interpersonal harm (e.g., accidents or illness) [24, 26]; thus, our discussion of threat focuses on interpersonal harm and violence.

### Threat/harm and underlying mechanisms

The central tenets of the DMAP suggest that early exposure to threat is primarily associated with alterations in socioemotional development that promote the rapid identification of threat cues in the environment, which in turn serves as a distinct pathway to the expression of psychopathology [4]. A seminal study pointing to differences in social information processing among individuals exposed to threat was conducted by Pollak et al. who examined different patterns of emotion recognition by maltreatment subtype [27]. Children exposed to threat showed a more liberal threshold for the detection of anger that was not present in control participants or those who experienced deprivation (i.e., neglect). Following this study, research investigating socioemotional differences among children exposed to ELA has tended to focus on specific maltreatment subtypes in isolation, rather than comparing similarities and differences between types of ELA. However, in studies investigating how threat exposure impacts socioemotional development, accumulating evidence supports the idea that children exposed to threat identify angry facial stimuli with less sensory information and with greater accuracy compared to healthy controls [28–33]. Pollak reported that children exposed to physical abuse were more accurate in identifying angry facial expressions, but not other emotions, compared to non-maltreated children [32]. This appears to be a dose-response relation, with greater exposure to abuse corresponding to quicker recognition of angry facial cues [33]. These results suggest that children exposed to threat have a perceptual bias *towards* the recognition of threatening stimuli in their environment, a conclusion further supported by studies demonstrating that children exposed to threat display an automatic attention bias towards threatening stimuli [34, 35]. However, it should be noted that there is heterogeneity around the directionality of this effect, with some studies reporting an attention bias *away* from threat cues associated with severity of exposure to threat in childhood [36]. Age may be an important moderator of these associations. For example, Weissman et al. showed that, as the severity of maltreatment increased, greater attention bias *towards* threat cues in children switched to a bias *away* from threat cues in older adolescents, thus underscoring the importance of developmental stage in examining links between threat exposure and attentional processes [37].

Although the ability to rapidly detect threat serves an adaptive function in dangerous environments, over time it may increase the risk of mental health difficulties. Previous studies have demonstrated associations between attentional bias to threatening cues and anxiety [38], depression [39], PTSD [40], and externalizing problems [41]. These attentional biases may relate to difficulties deploying effective strategies for regulating emotions, such as rumination and expressive suppression instead of adaptive skills like cognitive reappraisal [42]. Overreliance on unhealthy emotion regulation processes may increase transdiagnostic risk for psychopathology [43, 44]. Indeed, alterations in emotion regulation have been identified as an important mediator between childhood abuse and depression [45], self-harm [46], and PTSD [47]. Moreover, a recent study by Weissman et al. demonstrated that engagement in less optimal emotion regulation strategies, particularly expressive suppression and rumination, mediated the association between abuse exposure and longitudinal increases in general psychopathology (i.e., the “p” factor) [37].

Studies that have directly compared exposure to threat versus deprivation support the proposal that children exposed to violence and abuse have difficulties with socioemotional functioning that may uniquely increase the risk of psychopathology. For instance, some evidence suggests that children exposed to violence (a proxy for threat) show worse adaptation to emotional conflict after controlling for co-occurring poverty (a proxy for deprivation), yet the reverse effect is not significant [48]. Moreover, threat exposure has been shown to interact with age in predicting fear learning measured by physiological responses, whereas deprivation is unrelated to fear learning [49]. Finally, children exposed to threat may have distinct difficulties with social information processing. Prior research has shown that children exposed to threat are more likely to assume that ambiguous situations are hostile or dangerous [41, 50]. Information processing biases that result in the over-identification of situations as threatening may facilitate safety in dangerous environments, yet this developmental adaptation has also been associated with internalizing and externalizing problems [51]. A recent study found that differences in cognitive and affective theory of mind (ToM)—a key ability for social information processing—mediated the relation between interpersonal violence exposure and externalizing problems in adolescence [52]. This association remained significant after controlling for co-occurring deprivation (i.e., neglect), thus illuminating a unique pathway from threat exposure to externalizing symptoms. Given the above associations, it has recently been suggested that difficulties in social information processing and alterations in emotional processing are two core mechanisms linking threat exposure with negative mental health outcomes in children and youth [53].

### Threat/harm and neurobiology

Recent studies of children and youth exposed to abuse show clear associations between threat exposure and structural alterations in the frontoamygdala circuit, which is a cluster of brain regions important for threat detection and emotion processing. This includes reductions in amygdala volume [54–56] and the ventromedial prefrontal cortex (vmPFC) [57–59]. More negative coupling between the vmPFC and amygdala has also been observed in threat-exposed youth and is associated with higher externalizing symptoms [60].

Functionally, threat has also been shown to be associated with frontoamygdala circuitry. Studies have consistently found greater amygdala activity in youth who live in environments characterized by threat [61–64], although findings have been significantly more variable for vmPFC function [65]. Moreover, increased amygdala reactivity to negative stimuli may underlie risk of depression, anxiety, externalizing problems, and general psychopathology [66]. In contrast, a recent study conducted by Hein et al. reported decreased amygdala habituation (i.e., sustained activation) in a sample of 15–17 year olds exposed to threat when viewing angry facial stimuli, yet this was accompanied by low starting levels of amygdala activation, suggesting an overall blunting of amygdala activation [67]. This finding stands in contrast to the core hypotheses of the DMAP and requires replication in future longitudinal studies that track age-related changes in amygdala activation and habituation over time. In addition, threat exposure has been associated with differences in salience network activation. Studies measuring salience network activation have primarily reported increased anterior insula activation in response to threatening stimuli [61, 62, 68], while some studies report reduced dorsal anterior cingulate cortex (dACC) activation in response to threat cues [69]. It is worth noting here that these differences may be attributable to age-related changes in frontoamygdala communication. For example, recent work suggest that, among typically-developing youth, childhood may be characterized by information flow from amygdala to dACC to vmPFC (i.e., bottom-up processing), while adolescence may be characterized by information flow from vmPFC to dACC to amygdala (i.e., top-down processing) [70]. Further work should consider such developmental changes when interpretating the effects of ELA on neurodevelopment.

In terms of brain electrical activity (e.g., assessed using EEG), increased N2 and P3b activation has been observed in individuals exposed to threat as compared to controls when viewing angry faces, thus demonstrating a heightened vigilance for the rapid detection of threatening stimuli [31]. Further, this increased attention to threatening stimuli at the neural level mediated relationships between exposure to physical abuse and symptoms of anxiety [31].

Finally, a recent systematic review reported that alterations to the structure and function of the frontoamygdala circuit and salience network were consistently observed in children exposed to threat, but not necessarily those exposed to deprivation [65]. This adds further support for the differential neurocognitive consequences of threat compared to deprivation. An important next step for the field is to examine whether these neurocognitive differences mediate associations between unique dimensions of ELA and different forms of psychopathology. This requires longitudinal designs in which different dimensions of ELA are captured, different cognitive and neural phenotypes are measured, and multiple domains of psychopathology are jointly assessed. Especially important is the need for well-powered studies that can detect meaningful associations by avoiding false positive findings and failures of replication. An example of such a study is the Adolescent Brain Cognitive Development study [71], which will be an invaluable resource to the field in the years ahead.

## Deprivation

### Deprivation and psychopathology findings

Severe forms of psychosocial deprivation, such as institutional rearing, are associated with increased rates of multiple forms of psychopathology. By age 16 years, half of all children with a history of institutional deprivation meet criteria for any psychiatric disorder compared to only 15% of never-institutionalized youth [72]. The causal nature of this effect is suggested by studies showing that children randomly assigned to leave institutions early in development and enter family care have significantly fewer internalizing and externalizing symptoms than those with prolonged deprivation from childhood through adolescence [72–74]. For example, in the Bucharest Early Intervention Project (BEIP), youth with a history of prolonged institutional care had significantly higher rates of psychiatric disorders at age 16 years compared to those who left the institutions for foster care early in development [72]. The timing of intervention or removal from deprivation is also relevant. For example, individuals in the English and Romanian Adoptees (ERA) Study who were adopted before 6 months had similar levels of psychiatric symptoms to a group of non-deprived individuals, while those adopted after 6 months showed elevated rates of emotional difficulties, attention-deficit/hyperactivity disorder (ADHD), and neurodevelopmental impairments specifically associated with severe deprivation or insufficient caregiving (i.e., quasi-autism and disinhibited attachment) [75, 76]. Thus, existing evidence suggests that while there are early-emerging, significant, and in some cases persistent negative effects of institutional deprivation on mental health, enriched family care in the aftermath of deprivation can facilitate recovery, especially when it occurs early in development and is stable over time.

One domain of mental health that is strongly linked to early deprivation is ADHD. Multiple studies show that preschool-aged children reared in institutions have significantly higher rates of ADHD than never-institutionalized children [77, 78]. While the rates of many common psychiatric difficulties in children removed from institutions begin to approximate rates of never-institutionalized children by adolescence [72, 74], rates of ADHD generally persist through adolescence and young adulthood [72, 73, 75, 79, 80]. Some evidence suggests that those reared in institutions exhibit a distinct inattentive subtype of ADHD [80]. This may point to specific neurodevelopmental mechanisms impacted by early life deprivation (expounded below).

The link between deprivation and psychiatric difficulties is not limited to severe forms such as institutionalization. Indeed, other forms of deprivation, including poverty and low socioeconomic status (SES), are also associated with increased rates of psychopathology [81, 82]. Furthermore, previous reviews and meta-analyses have shown that different types of maltreatment (abuse and neglect) are associated with greater risk for multiple mental health problems [83–85]. While there is some variability in the extent of risk conferred by specific types of ELA, experiences of both threat (e.g., abuse) and deprivation (e.g., neglect) show significant positive associations, with largely similar effect sizes [83–87]. Thus, each of these dimensions is generally considered a non-specific risk factor for mental health problems. In contrast, there is evidence that deprivation is associated with partially distinct mechanisms that underlie psychopathology compared to threat. We explicate these mechanisms below.

### Deprivation and underlying mechanisms

The DMAP suggests that early deprivation is primarily associated with alterations in cognitive functioning that are not observed as consistently among those exposed to threat [4]. In support of this hypothesis, exposure to deprivation has been consistently linked to a range of cognitive and academic difficulties [88–93]. It has also been shown that early intervention can offset these cognitive difficulties through early removal from institutions and placement in high-quality family care settings [88, 89, 94, 95]. Early work by Tizard and Rees demonstrated that improving the quality of institutional care to focus on providing enriched social and cognitive stimulation can mitigate cognitive impairments associated with institutionalization [96]. Indeed, a core mechanism underpinning the cognitive difficulties associated with early deprivation is in the lack of socioemotional and cognitive input that characterize both institutional care and family-based neglect, the most common form of maltreatment in the United States [97]. Thus, the difference between institutional deprivation and common forms of neglect is likely a matter of degree rather than a qualitative difference.

In addition to impairments in general intellectual and academic abilities, deprivation has also been associated with reduced language skills [90, 91, 98, 99]. In studies of typically-developing children, reduced language input from caregivers at 18 months is associated with lower pre-academic skills at age 4.5 years through a host of cognitive skills at age 3, including reduced language ability, inhibitory control, and ToM [100]. Interestingly, when controlling for maternal linguistic input (i.e., stimulation), maternal responsiveness (e.g., warmth and sensitivity) was not strongly associated with these cognitive abilities. Recent meta-analytic work has shown a moderate inverse association between experiences of both threat and deprivation with language abilities, but no moderating effect of ELA type [101]. In contrast, recent longitudinal work has reported that while both threat and deprivation in childhood are associated with increased risk of internalizing and externalizing problems in adolescence, only deprivation operates through language ability [102, 103]. Evidence from preschool children similarly shows that lower SES (a proxy for deprivation) is associated with reduced language ability at age 3, and that lower language ability is correlated with higher general psychopathology at age 4.5 years [104]. Given the importance of language abilities for self-regulation, effective problem-solving, and emotional expression and awareness, this may serve as a key mechanism connecting early deprivation to broad-spectrum psychopathology over the course of childhood and adolescence.

Another domain of cognition consistently associated with early deprivation is executive function (EF). Per the DMAP, reduced EF is a distinct neurodevelopmental mechanism linking deprivation with psychological functioning. In support of this claim, reductions in EF—specifically response inhibition and working memory—have been shown to mediate the association between institutional deprivation and ADHD symptoms in childhood (ages 8 and 12 years) [105, 106]. Moreover, children who experience less severe forms of deprivation, such as low SES, show increased rates of ADHD [107]. Interestingly, in longitudinal studies that include measures of threat and deprivation simultaneously, only deprivation has been shown to mediate the effect of low SES on poor EF outcomes in preschool-aged children [108]. However, the mediational role of EF between deprivation and psychopathology is not specific to ADHD, with recent evidence suggesting that reduced EF in mid-childhood is a transdiagnostic mediator of psychopathology risk in adolescence [109]. These results together suggest that both common (e.g., low SES) and severe (e.g., institutional rearing) forms of deprivation are associated with EF difficulties that increase the risk of psychopathology in multiple domains.

Perhaps the best evidence for the differential effect of deprivation relative to threat on EF comes from studies that include both dimensions in the same models. Using this approach, Sheridan et al. showed that, when controlling for experiences of threat (i.e., abuse and community violence), experiences of deprivation (i.e., neglect) were associated with lower parent-reported inhibition and global EF, while exposure to threat was unrelated to EF after controlling for deprivation [110]. Using objective measurement of EF, it was further shown that lower parental education (a proxy of deprivation) was associated with reduced working memory performance after controlling for exposure to threat, while threat was not significantly associated with working memory after controlling for deprivation. Another study found that children who experienced deprivation (operationalized as living below the federal poverty line) showed significant impairment in inhibition and shifting ability, which was not observed among children exposed to threat [48]. A more recent study by Machlin et al. found that a composite measure of deprivation―neglect, parental education, and household stimulation—was associated with impairments in response inhibition after controlling for exposure to threat [49]. Finally, in a recent study using a representative sample of >18,000 U.S. Kindergarten students, cumulative exposure to deprivation, but not cumulative exposure to threat, was associated with lower working memory, while both cumulative deprivation and cumulative threat predicted lower cognitive flexibility and inhibitory control [111]. One explanation for these mixed findings may be the use of objective EF assessment for cognitive flexibility and working memory, but teacher reports for inhibitory control in the latter study. It is possible that a blunt tool such as teacher ratings is not sensitive enough to detect subtle differences in risk conferred by different types of ELA. In contrast, clearer distinctions in the risk of EF difficulties may emerge when using objective assessments. To this end, the results noted above cohere with recent meta-analytic work showing that early life deprivation has stronger associations with working memory and inhibitory control than early life threat, while both types of ELA are approximately equally related to cognitive flexibility [112].

It has been suggested that deficits in associative learning may serve as a common mechanism linking deprivation to multiple domains of cognitive functioning [113]. The framework for this model is derived from the notion that associative learning promotes the formation of new synaptic connections, yet in the absence of contingent responsiveness (e.g., from caregivers) or a general lack of available rewards in the environment (e.g., provision of food, physical proximity, and affection), synaptic connections will either not form (i.e., reduced proliferation) or will be prematurely eliminated (i.e., accelerated pruning). Support for this model comes from studies looking at alterations in stimulus-response relationships, with consistent findings that children reared in institutions perform significantly worse than never-institutionalized children on associative learning tasks [114–116]. In turn, reduced associative learning mediates the relation between early deprivation and depression symptoms in early adolescence [114]. Strikingly, studies also show that children removed from institutional settings at early stages of development perform as well as children who were never institutionalized on associative learning tasks [114, 115], suggesting that recovery is possible when contingent responsiveness is restored. It is important to note, however, that deficits in associative learning have also been linked to early threat exposure [117, 118]. However, to our knowledge no study has examined the association between threat and deprivation with associative learning while controlling for the effect of the other dimension of ELA, which is required to determine specificity of these associations. This is an important direction of future work.

### Deprivation and neurobiology

Evidence from developmental cognitive neuroscience demonstrates clear associations between early deprivation and brain structure and function in regions involved in EF. Structurally, deprivation is correlated with reduced gray matter in the prefrontal, parietal, and temporal regions, as well as reductions in total gray matter and white matter volume [119–121]. A recent systematic review by McLaughlin et al. [65] highlighted that deprivation is associated with fairly reliable reductions in volume or thickness of frontoparietal network regions, including the dorsolateral PFC (dlPFC) and superior parietal cortex. Altered structure in these areas is, in turn, linked with increased risk for multiple types of psychopathology [122, 123]. Some evidence for alterations in structural connectivity also exist, with reduced fractional anisotropy (FA; measuring white matter microstructural organization) in the superior longitudinal fasciculus and inferior fronto-occipital fasciculus in relation to deprivation [124, 125]. This is in contrast to threat exposure, where *increased* FA has been observed in relation to traumatic events [126].

Furthermore, altered neural function of frontoparietal regions and other regions important for EF have been observed in youth exposed to deprivation. For example, on a cognitive task involving neutral stimuli, children exposed to deprivation recruited the inferior frontal cortex and dACC more strongly than control subjects [127]. Additionally, in a recent study by Silveria et al. functional connectivity of the dACC to other regions within the cingulo-opercular network were found to mediate the association between deprivation and externalizing symptoms [128]. Furthermore, on cognitive control tasks involving emotional cues, children exposed to deprivation show reduced recruitment in the dlPFC [129]. This is in contrast to children exposed to threat, who have been shown to demonstrate *increased* recruitment of the PFC during cognitive control of emotion [61, 65]. Again, this suggests differences in how cognitive control is deployed to regulate emotionally-salient cues based on the dimension of ELA.

Consistent with functional findings, studies have demonstrated associations between early deprivation and disruptions in brain activity within regions directly involved in cognitive control. Studies examining neural indices of cognitive control, as measured by event-related potentials (ERPs), have found that reduced (less negative) amplitude of ERPs is linked with increased externalizing and ADHD symptoms in institutionalized youth [130, 131]. Furthermore, task-evoked mediofrontal theta power during response inhibition has been shown to mediate the association between deprivation and general psychopathology in adolescence [132]. Similar to brain structure, alterations in neural activation across regions implicated in cognitive control have been associated with psychopathology transdiagnostically [123, 133].

## Unpredictability

The third and final dimension of ELA we cover in this review is unpredictability, which is believed to differ in its developmental consequences compared to harshness/threat [13]. According to Life History (LH) theory, in *harsh* environments there is less access to necessary resources and a correspondingly greater risk of external injury, illness, or death. In contrast, *unpredictability* refers to stochastic fluctuations in harshness over space and time (i.e., residential transitions, how often caregivers change jobs, and changes in cohabitation) [134]. An outgrowth of the LH model is the Adaptive Calibration Model (ACM), which postulates that individual differences in physiological stress responsiveness and related behaviors are a consequence of adaptation to the conditions of the environment [135]. This means that stress response systems of an organism mediate alternate LH strategies as a function of exposure to threatening or unpredictable environments. The ACM is primarily focused on stress-mediated behaviors that relate to growth and reproduction, including exploration, learning, parenting, risk-taking, impulsivity-aggression, and cooperation [136]. Another recent model that has emerged from LH theory which more explicitly focuses on cognitive functions associated with adversity is the Hidden Talents Approach [137]. This approach is unique in highlighting cognitive advantages conferred by adversity that permit adaptation to threatening and/or unpredictable environments. The Hidden Talents Approach, like the DMAP, acknowledges that specific experiences and exposures may have distinct associations with certain cognitive abilities because they pose different developmental challenges [138]. This model grants its own strengths and limitations, including issues related to measurement and how to differentiate between dimensions of ELA [138]. Notably, the challenge of disaggregating dimensions of ELA is not specific to the Hidden Talents Approach, as adversities indeed co-occur and can make it difficult to model ELA dimensionally [20]. However, this problem is not so difficult that it cannot be overcome with adequate sampling and statistical techniques [18]. A related conceptual difficulty is disambiguating unpredictability from deprivation. Both unpredictability and deprivation relate to socioeconomic disadvantage, which is often used as a proxy for both dimensions in the extant literature. Yet another question that requires further inquiry is how the dimension of unpredictability differs from, or includes, related constructs such as household, school, or neighborhood chaos or instability. These issues are reviewed in detail elsewhere [138, 139]. For the purpose of the current review, we include chaos and instability within the general construct of unpredictability.

### Unpredictability and psychopathology findings

Although LH theory and the Hidden Talents Approach posit individual strengths that may arise from ELA, other studies have noted that unpredictable environments are associated with increased risk for mental health problems in children and youth [140, 141]. For example, externalizing behaviors at age 16 have been found to mediate the link between early unpredictability and externalizing and criminal behaviors in young adulthood [142]. Moreover, in a sample of college students, childhood unpredictability with respect to meals, money, discipline, and nurturance was associated with more symptoms of depression and anxiety [143]. In a sample of adolescents, unpredictability (measured as parental inconsistency) was shown to correlate with general psychological disorder symptoms [144]. Studies of school-age children have similarly shown that the effects of low SES on socioemotional problems is mediated by heightened levels of chaos, operationalized as noise, crowding, foot traffic, confusion at home, and disrupted routines and rituals [145]. In other studies of young children, household chaos has been associated with more conduct problems even after controlling for socioeconomic factors [146]. Moreover, when dimensions of both household instability (e.g., number of moves, changes in caregiver) and household disorganization (e.g., cleanliness, neighborhood noise) are measured in early childhood, disorganization, but not instability, has been associated with child conduct problems and callous-unemotional traits in first-graders [147]. Also interesting in the latter study is that both disorganization and instability were associated with higher callous-unemotional traits through increased parental harshness. As with studies drawing on the DMAP dimensions of threat and deprivation, these findings underscore the non-specificity of unpredictability with respect to psychopathology, instead suggesting that unpredictability may be a transdiagnostic risk for both internalizing and externalizing problems.

### Unpredictability and underlying mechanisms

As noted above, unpredictability is a dimension of ELA that is particularly difficult to measure, with many heterogeneous approaches [139]. Despite this caveat, there is some evidence linking environmental unpredictability to cognitive mediators of psychopathology. Perhaps the strongest evidence for cognitive alterations associated with exposure to household chaos comes from a recent meta-analysis which showed that disorganization and instability in the home have small but significant associations with EF [148]. The effects were stronger for informant-completed reports than direct assessment of EF, and for instability compared to disorganization dimensions of chaos. These results dovetail with the recent meta-analysis reported above in which both dimensions of threat and deprivation were associated with reductions in certain EF domains, despite stronger associations for deprivation [112].

Notwithstanding the evidence linking dimensions of threat, deprivation, and unpredictability to both heightened psychopathology and reduced EF, LH models propose that early exposure to harsh and unpredictable environments involves developmental tradeoffs that promote traits synonymous with a “fast lifestyle” which may be beneficial in meeting the challenges of stressful and unpredictable environments [13, 137, 149]. One such adaptation is an enhanced ability to flexibly switch between tasks or mental sets and for tracking novel environmental information, especially under conditions of stress or uncertainty. Indeed, there is some evidence of enhanced cognitive flexibility (or shifting of attention) [150, 151] and updating working memory [152] among those exposed to early unpredictability. Most of these studies have been conducted with adults who retrospectively report on childhood unpredictability, yet some evidence has recently linked unpredictability—measured by the number of paternal transitions—to improved effortful control in preschool children [153]. Likewise, caregiving instability defined as the number of caregiver switches has recently been associated with lower response inhibition and attentional control, but enhanced cognitive flexibility in school-aged children [154]. It is suggested that these developmental enhancements may reflect a process of adaption to meet the challenges associated with unpredictable early environments. Although these observations seem relatively specific to experiences of unpredictability, recent evidence has also linked early deprivation to enhanced working memory [155]. Determining the conditions under which decrements versus enhancements in specific aspects of EF emerge, and the implications of this for mental health, remains an important area for future work in this field.

### Unpredictability and neurobiology

Research investigating unpredictability suggests that there are neurobiological adaptations to unpredictable environments [137]. However, it is not yet clear how or if these adaptations differ from those that result from other dimensions of ELA (i.e., threat and deprivation). Differences in amygdala volume and reactivity, as well as functional connectivity of brain circuits involving the PFC and amygdala, have been cited as possible neurobiological adaptations arising from unpredictable or harsh environments [137]. Importantly, these alterations have also been associated with exposure to other types of ELA (see above). A limited number of studies have directly examined the effects of unpredictability on neural development (see Liu and Fisher [156] for a review). For example, infant exposure to unpredictable maternal sensory signals (e.g., visual, auditory, and tactile inputs during free play) has been associated with greater integrity in the uncinate fasciculus but not the hippocampal cingulum [157]. Moreover, the ratio of uncinate fasciculus to hippocampal cingulum generalized FA mediated the associated between maternal unpredictability and performance on an episodic memory task. It has also been shown that children aged 8–10 years old demonstrate heightened amygdala responses to unpredictable threat compared to neutral cues during an fMRI-based task [158].

Furthermore, high levels of unpredictability are associated with brain functional alterations in regions subserving cognitive control. For instance, household chaos has been shown to moderate the association between parental behavioral control and activation of the insular cortex—a region involved in salience processing and often considered part of a wider network of cognitive control, including flexibility [159]—in a sample of adolescents [160]. Household chaos has also been shown to moderate the association between parental control and a global factor indexing neural activation during cognitive control in several regions, including the insula and regions comprising the frontoparietal network (e.g., inferior and middle frontal gyrus) in a cohort of adolescents [161]. Specifically, only in low chaos homes was higher parental control associated with better neural cognitive control among adolescents. However, household chaos was not directly related to neural cognitive control. This suggests that the ability of parents to serve as effective regulators of adolescent’s cognitive control may be undermined in high chaos environments. In turn, poor neural cognitive control was associated with compromised social competence only in high chaos households. These results highlight the fact that different dimensions of ELA may not only directly impact cognitive control and its underlying neural mechanisms, but may shape the way in which the environment scaffolds these skills, including how responsive children and youth are to social organizers of cognition and emotion, ultimately increasing the risk of psychopathology and other functional outcomes.

## Evidence directly supporting dimensional models of ELA

It is important to note that very few studies have studied the unique effects of threat/harshness, deprivation, and unpredictability *simultaneously* in relation to mental health, cognition, and/or their neurobiological underpinnings. As such, this section is dedicated to outlining findings from studies that have taken an integrated dimensional approach. One such study measured exposure to violence (threat), cognitive stimulation (deprivation), and unstable aspects of the physical environment such as cleanliness and safety (unpredictability) [162]. Assessments of associative memory, cued attention, and memory-guided attention were conducted. The results showed that violence exposure was specifically associated with poor associative memory after controlling for cognitive stimulation and the physical environment. In contrast, cognitive stimulation and the physical environment were associated with cued attention, but not independently of other environmental factors. Finally, the physical environment was associated with memory-guided attention after adjusting for violence and stimulation. While not neatly mapping onto hypotheses derived from DMAP or LH models, these results suggest differential associations between different dimensions of ELA and specific cognitive abilities. An important next step in this work is to determine whether these cognitive alterations mediate psychopathology risk in children and youth following exposure to specific dimensions of ELA.

To our knowledge, only one study has simultaneously measured the neurobiological effects of threat, deprivation, and unpredictability over time [163]. In this recent three-wave longitudinal study of 9–19-year-old participants, higher family-level deprivation was broadly associated with lower initial resting-state connectivity and less connectivity increases over time both within networks (salience network and default mode network) and between networks (default mode-to-frontoparietal, default mode-to-salience, and frontoparietal-to-salience networks). Neighborhood deprivation was not associated with any specific connectivity differences. Adolescents exposed to unpredictability had lower initial levels of salience network connectivity and had less of a connectivity increase over time between both salience and default mode networks, as well as between salience and frontoparietal networks. Adolescents exposed to threat demonstrated slower decreases over time within frontoparietal networks and between default node and salience networks. The authors concluded that, while family-level deprivation may diffusely impact neural connectivity across multiple networks, both threat and unpredictability may have more focal connectivity correlates. These results demonstrate that different dimensions of ELA may be associated with different underlying neurobiological mechanisms. However, it should be noted that there is some variability within recent longitudinal studies examining connectivity differences across dimensions of adversity. For example, one study reported that salience network resting-state functional connectivity tended to increase for both individuals exposed to threat and deprivation in a longitudinal sample of adolescents aged 16–19 years, and these increases mediated associations between different dimensions of ELA and depressive symptoms at age 19 years [164]. Another longitudinal study of adolescents aged 15–17 years reported reduced resting-state functional connectivity density within the salience network, and between default mode and salience networks, among those exposed to threat, but not social deprivation [165]. It is possible that these study differences in connectivity are attributable to age-related changes in connectivity, the timing of adversity exposure, or other sampling/measurement factors that make clear conclusions difficult to draw. Additional studies that catalog connectivity differences from childhood to through adolescence will be required to better understand these findings.

Finally, we would like to highlight a recent large-scale longitudinal study by McGinnis et al. (2022) which draws attention to interpersonal loss (e.g., death of a parent) as another potentially distinct dimension of ELA alongside the existing dimensions of threat, deprivation, and unpredictability [166]. Consistent with the core hypotheses of the DMAP, McGinnis et al. found that only material deprivation was associated with the proximal outcome of lower IQ in early adulthood despite being associated with a host of later psychiatric and functional outcomes, lending support to alterations in cognitive function as a putative mechanism linking deprivation to broad psychological well-being. Moreover, although no proximal outcomes associated with emotional functioning were evaluated within this study, threat was the only dimension of ELA that was significantly correlated with both adult depressive and anxiety as distal psychiatric outcomes, lending some support to alterations in emotional functioning following threat exposure. Moreover, unpredictability was associated with number of sexual partners at age 16 and downstream conduct and criminality outcomes in adulthood, consistent with LH theory. Uniquely, interpersonal loss was not associated with any psychiatric outcome or cognitive function in adulthood, but was associated with both sexual partners at age 16 years and age at first childbirth, as well as economic and conduct problems as functional outcomes in adulthood. Interestingly, after controlling for the dimensions of adversity noted above, a composite measure of “other adversities”—which included exposure to illness, disaster, and accidents—was associated with only one-of-four psychiatric outcomes (cannabis use), as well as distal health, economic, and conduct problems, but was unrelated to any proximal mechanism proposed by either DMAP or LH theory. The fact that each of the four dimensions of ELA assessed (threat, deprivation, unpredictability, and loss) was associated with multiple psychiatric and functional outcomes but partially distinct proximal mechanisms lends support to dimensional models of ELA. Indeed, future longitudinal work that examines other potential dimensions of adversity in addition to those noted above will improve our understanding of the shared and distinct consequences of ELA and their associated mechanisms.

## Conclusion and future directions

Emerging evidence supports the use of dimensional models in mapping the cognitive, socioemotional, and mental health consequences of ELA, with partially distinct neurodevelopmental mechanisms operating for different dimensions of ELA. Specifically, while the dimensions of threat/harshness, deprivation, and unpredictability appear to confer increased risk for multiple mental health difficulties along the internalizing and externalizing spectra, they do not necessarily do so via the same neurocognitive and socioemotional pathways. However, this is not to say that the mechanisms connecting ELA to psychopathology are completely distinct. Indeed, while some meta-analyses show significant associations for one dimension of ELA but not the other (e.g., associations of threat but not deprivation with markers of biological aging [167]), others show this distinction only in degree (e.g., both threat and deprivation are associated with EF, with stronger associations for deprivation [112]). There are also some aspects of functioning or physiology that are common across dimensions of ELA, such as stress system responsiveness [18].

With an eye to the future, the field will likely begin to focus on the ways in which multiple transdiagnostic mechanisms work in tandem to heighten the overall risk of psychopathology. In the case of co-occurring adversities across different dimensions of ELA, it will be important to examine how the cognitive and socioemotional difficulties inherent to threatening, depriving, and unpredictable experiences interact with one another to shape mental health outcomes. Indeed, in the cumulative risk literature it is well-established that as risks accrue, so too does the risk of psychopathology [168]. While frequently attributed to processes such as increased allostatic load [169], it may also be that exposure to multiple adversities up- or downregulates several neurodevelopmental processes that potentiate one another to exacerbate psychopathology risk. To this end, a recent study investigating white matter connectivity differences among adolescents who experienced adversity in childhood reported that, although there were no specific differences been individuals who experienced threat compared to social deprivation, there was a significant interaction between exposure to violence and social deprivation such that violence exposure only predicted white matter differences when social deprivation was high, but not when social deprivation was at mean or low levels [170]. This study illustrates that multiple co-occurring adversities or singular exposures can be studied through a dimensional lens to more comprehensively understand how dimensions of ELA are associated with the neurobiological mechanisms underlying psychopathology in childhood and adolescence.

Moreover, in the case of complex exposures (e.g., poverty, homelessness, etc.), multiple or singular mechanisms may account for the increased psychiatric risk, depending on the specific exposures of a given child in those settings. For some children and adolescents, this may mean experiencing predominantly threat (e.g., sexual abuse/exploitation), and for others it may mean deprivation (e.g., isolation) or unpredictability (e.g., inconsistent access to shelters or food). However, the fact that complex exposures may involve multiple ELA dimensions does not mean we should expect that each of these dimensions is the same with respect to their developmental consequences; and indeed, each person is likely to be differentially impacted based on their unique clustering of adversities within a given exposure. This is why dimensional models advocate for a more nuanced assessment of what specifically is occurring within a given exposure that signals the presence of threat, deprivation, or unpredictability. In contrast, cumulative adverse childhood experience (ACE) models—which simply sum adversities together—assume each ACE operates on the same mechanism and is weighted equally [18]. While such approaches may be useful in public health initiatives to screen for broad risk (though see Finkelhor for cautions to this approach, and Baldwin et al. for evidence of poor accuracy in predicting individual risk from ACE scores), cumulative ACE models are less helpful in ascertaining the mechanisms by which adversity confers increased mental health risk [171, 172]. This highlights a need for improved measures that can help to chronicle various ELA types and their underlying dimensions to better understand the mechanisms involved in the sequelae of adversity [173]. Applied widely, use of such measures will help to control for methodological differences that may explain heterogeneity in the pattern of results across studies, thus aiding replicability and generalizability within the field of adversity science.

We believe that an integrated framework is required in order to better understand the shared and distinct outcomes and mechanisms associated with ELA along the dimensions of threat/harm, deprivation, and unpredictability [18], as well as lesser-studied dimensions (e.g., loss). This includes theoretical models that can be explicitly tested and which draw on multiple perspectives in psychology, neuroscience, cognitive science, psychophysiology, and evolutionary theory, as well as the development of new tools for better assessing and distinguishing between dimensions of ELA in diverse populations. Programs of research that assess multiple types of ELA and which simultaneously chart functioning in numerous neurocognitive and socioemotional domains should be prioritized to further determine the validity and utility of dimensional models, with concerted efforts to create cohesion in the measures and methods used to allow for joining of datasets and to facilitate future meta-analytic work. It may be too early to determine how dimensional models can inform clinical initiatives that tailor interventions to the unique needs of individuals, but such an approach is consistent with parallel initiatives in psychiatry and neuroscience which aim to better characterize the mechanisms underpinning risk of psychopathology. This includes the recently developed Hierarchical Taxonomy of Psychopathology (HiTOP) as a dimensional classification system for mental health problems, and the Research Domain Criteria (RDoC) as a research framework for identifying transdiagnostic biobehavioral systems implicated in psychopathology [174]. Despite the potential of these complementary approaches to inform the development of a dimensional psychiatric nosology, neither of them explicitly includes ELA as a target of assessment or measurement. However, dimensional models of ELA clearly square with both the dimensional and mechanistic focus of HiTOP [175] and RDoC [176]. We believe that further integration of dimensional models of ELA—one of the most robust risk factors for psychopathology—into these models will advance efforts to improve how we study, assess, diagnosis, and ultimately treat mental health problems in children and youth.
